# Dual control by a single gene of secondary sexual characters and mating preferences in medaka

**DOI:** 10.1186/1741-7007-7-64

**Published:** 2009-09-29

**Authors:** Shoji Fukamachi, Masato Kinoshita, Kouichi Aizawa, Shoji Oda, Axel Meyer, Hiroshi Mitani

**Affiliations:** 1 Department of Biology, University of Konstanz, D-78457 Konstanz, Germany; 2 Department of Integrated Biosciences, University of Tokyo, Chiba 277-8562, Japan; 3 Division of Applied Biosciences, Graduate School of Agriculture, Kyoto University, Kyoto 606-8502, Japan

## Abstract

**Background:**

Animals utilize a wide variety of tactics to attract reproductive partners. Behavioral experiments often indicate an important role for visual cues in fish, but their molecular basis remains almost entirely unknown. Studies on model species (such as zebrafish and medaka) allow investigations into this fundamental question in behavioral and evolutionary biology.

**Results:**

Through mate-choice experiences using several laboratory strains of various body colors, we successfully identified one medaka mutant (*color interfere*; *ci*) that is distinctly unattractive to reproductive partners. This unattractiveness seems to be due to reduced orange pigment cells (xanthophores) in the skin. The *ci *strain carries a mutation on the *somatolactin alpha *(*SLa*) gene, therefore we expected over-expression of *SLa *to make medaka hyper-attractive. Indeed, extremely strong mating preferences were detected in a choice between the *ci *and *SLa*-transgenic (Actb-SLa:GFP) medaka. Intriguingly, however, the strains showed opposite biases; that is, the mutant and transgenic medaka liked to mate with partners from their own strain, similar to becoming sexually isolated.

**Conclusion:**

This study spotlighted *SLa *as a novel mate-choice gene in fish. In addition, these results are the first demonstration of a single gene that can pleiotropically and harmoniously change both secondary sexual characters and mating preferences. Although theoretical models have long suggested joint evolution of linked genes on a chromosome, a mutation on a gene-regulatory region (that is, switching on/off of a single gene) might be sufficient to trigger two 'runaway' processes in different directions to promote (sympatric) speciation.

## Background

Reproduction is one of the most important events in life. Animals of many species try a wide variety of measures, such as songs, dances, scents, ornaments, gifts, or electric fields [1], to attract mating partners for successful reproduction. Researchers have found that pre-mating sexual isolation by mate choice (that is, not by incompatibility of genital or other morphologies for copulation [2,3]) exists in various animal taxa including yeasts [4]. Teleost fish have also been used frequently as models for mate-choice experiments [5], and visual cues have often been shown to play an important (but not exclusive) role in mate attraction (for example, body colors, fin shapes, courtship displays, and so on). However, the cellular/molecular basis of visual-based mate choice remain largely obscure, whereas those of the olfactory/auditory-based mate choices are understood in greater detail for model organisms such as mice, fruit flies, or nematodes [6-8].

Body colors are rapidly evolving, so that highly divergent traits of animals and distinct color variants often occur even within a species, including humans. Although many such variants are post-zygotically compatible (that is, inter-variant hybrids become viable and fertile), they sometimes prefer intra-variant mating to inter-variant mating. This mutually exclusive reproduction (assortative mating) suppresses gene flow between the variants, and is thought to drive speciation in the absence of geographical barriers (that is, sympatric speciation [9] by sexual selection).

Hence, an important question in behavioral and evolutionary biology is how is such intra-variant mating controlled? That is, what genes function and how do they evolve to shape 'harmoniously polymorphic' secondary sexual characters and mating preferences? Theoretical prediction and limited empirical evidence support the joint evolution of two or more genes linked on a chromosome that control either secondary sexual characters or mating preferences [10-12]. However, the specific genes responsible (particularly, those for mating preferences) are yet to be identified. Hence, adequacy of a model assuming such convenient mutations with harmonious *in-vivo *effects that simultaneously occurred on a chromosome (or somewhere in a genome which consequently developed physical linkage or linkage disequilibrium) remains unclear. Perhaps a simpler mechanism is more likely: that a single mutation on a gene that has pleiotropic functions harmoniously alters both secondary sexual characters and mating preferences (see [13]).

**Figure 1:**
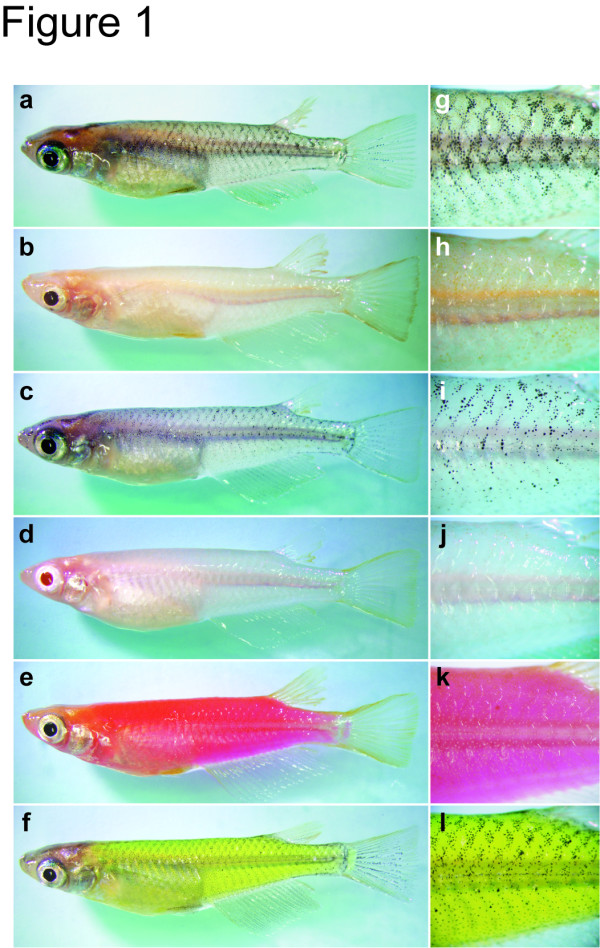
**Medaka mutant/transgenic strains tested in this study**. **(a) **Wild type (HNI); **(b) ***b*^*g8*^; **(c) ***ci*; **(d) **albino (*i*^3^); **(e) **OlMA1-DsRed2; **(f) **OlMA1-GFP; **(g-l) **larger magnification of a-f. Genetic backgrounds and detailed phenotypes of these fish are described elsewhere [18,20,40,41].

Medaka, *Oryzias latipes*, is an excellent organism for functional genetics but knowledge of its secondary sexual characters and mating preferences is poor. As in many other vertebrates, medaka females (and also males) prefer mates with a larger body size, probably because it correlates with greater fecundity and reduces the risk of gamete depletion [14,15]. Males have slightly longer dorsal/anal fins (which should mechanically help to hold a spawning female; see *Results*) and more pigmented caudal/anal fins [16]. These potentially sexual characters are, however, far less pronounced than those of many other fish, such as the caudal fin of swordtails or body colorations in guppies. This inconspicuous sexual dimorphism in medaka has made it difficult to design mate-choice experiments. Nevertheless, once the secondary sexual characters for medaka are identified, this model species will provide a powerful and representative platform for studying cellular/molecular basis of the visual-based mate choice in fish. Based on these considerations, we ask whether or not medaka prefers a certain body color using various mutant and transgenic strains (Figure 1).

## Results

### Medaka mating behaviors and experimental design of mate choice

The feature that makes medaka a convenient candidate for studying mate choice is that they spawn every morning. Therefore observation of their behaviors in the early morning should facilitate reliable evaluation of mating (but not association/schooling) preferences. Typical mating occurs as follows: (1) a male rapidly comes close to and takes/keeps a position beneath a female, (2) he performs the 'round dance' (that is, swimming in a rapid circle [14,15,17]) in front of her and gets back to the position, which is often repeated or omitted, (3) he comes up next to and holds on to her using his dorsal/anal fins, (4) when she accepts him, she slightly pushes him aside, and then spawns her eggs lasting typically about 20-30 seconds [see Additional files 1 and 2].

**Figure 2:**
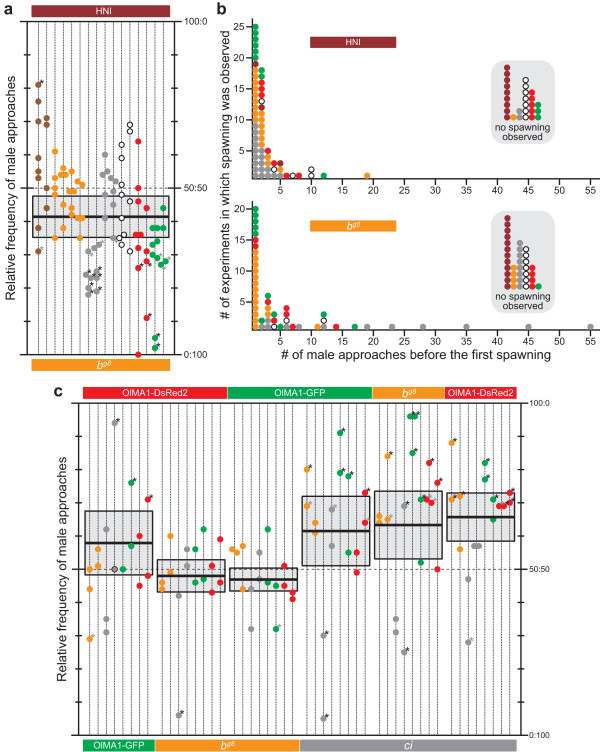
**Screening for potentially attractive/unattractive strains**. (a) Male mating preferences between HNI and *b*^*g8 *^females. The choice females are indicated on top and bottom (*n *= 16; 8 for each). Dotted vertical lines represent individual test males (*n *= 16), and each circle on the lines shows a result from each mate-choice experiment (4-8 experiments for each male; see *Methods*). Colors indicate strains: HNI (brown), *b*^*g8 *^(orange), *ci *(gray), *i*^3 ^(white), OlMA1-DsRed2 (red), and OlMA1-GFP (green). The shaded box with a horizontal bar shows the mean and 95% confidence limits. An asterisk indicates that male approaches in the experiment were not random (50:50) according to the two-tailed binomial test (gray, *P *< 0.01; black, *P *< 0.001). (b) Female mating preferences. The identical 88 experiments in Figure 2a were differently analyzed to evaluate female mating preferences (upper, HNI; lower, *b*^*g8*^). Colors of circles indicate strains of males with which the females spawned. (c) Male mating preferences between females of various strains. Males (*n *= 8; 2 each from *b*^*g8*^, *ci*, OlMA1-DsRed2, and OlMA1-GFP strains) were given choices of all female combinations of the four strains (*n *= 16; 4 for each strain). Note that the 95% confidence limits do not include 50:50 only when *ci *is used as a choice female. *ci *females are significantly less attractive than *b*^*g8 *^and OlMA1-DsRed2 (but not OlMA1-GFP) females according to the one-way repeated measures ANOVA and the post-hoc least significant difference (LSD) test (*F*_3,21 _= 5.197, *P *= 0.008; see *Methods*).

When we put one male and two females in a tank and let them freely mate for one hour, males vigorously approach both females regardless of whether the females are gravid or have already spawned (for example, 63 ± 2.1 approaches/hour in 188 experiments in Figure 2; mean ± SEM). Male approaches to spawned females are not always fruitless because females sometimes spawn multiple times in a morning (for example, we observed it in 28 out of 236 matings in Figure 2). Thus, we counted the number of male approaches to each female (defined as jerking towards a female and positioning beneath her) as a sign of his mating preferences, regardless of whether the female remained in position or tried to escape, as we occasionally observed.

By contrast, females very rarely approached males, or interacted with other females (for example, fighting for males) under this free-swimming condition (see also [14]). Given the frequent approaches from males (see above), females appeared to stay still when ready for spawning. Nevertheless, we observed female approaches in extremely rare cases; when males are indifferent about mating (which is already an exceptional case), females performed the 'round dance', which has been regarded as a male-specific courtship display. Although the finding is interesting, the female's dance seems to be too infrequent to be statistically analyzed (we observed only a few dances during a total of >400 hours of observations). Hence, we counted the number of male approaches until each female spawned, with the expectation that this inverse index could reflect mating preferences of these intrinsically passive medaka females (that is, fewer approaches, more preferable).

**Figure 3:**
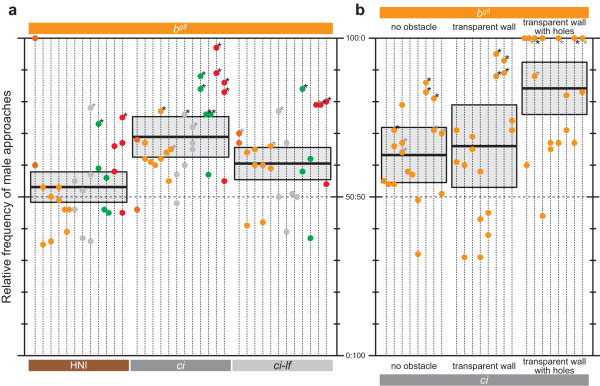
**Dissection of the sensory cue that causes male's sexual discrimination against *ci***. **(a) **Males' discrimination against xanthophore-less females. Males of various strains (*n *= 12) were offered three female combinations of *b*^*g8 *^and either of HNI, *ci*, or *ci-lf *(*n *= 18; 6 for each strain). Note the biased preferences in the choices of *b*^*g8*^-*ci *and *b*^*g8*^-*ci*-*lf*. The one-way repeated measures ANOVA and the post-hoc LSD (but not Bonferroni) test, however, detected significant difference even between these choices (*F*_2,22 _= 16.730, *P *< 0.001; post-hoc LSD, *P *= 0.022; post-hoc Bonferroni, *P *= 0.067). Hence, the increased leucophores may additively contribute to the unattractiveness of *ci*. **(b) **Male's sexual discrimination against *ci *recreated under non-free-swimming conditions. Males of *b*^*g8 *^(*n *= 8) were presented choice females of *b*^*g8 *^and *ci *(*n *= 16; eight for each strain) either under the free-swimming conditions, in flasks, or in holed flasks.

Identification of the unattractive mutant, color interfereIn order to screen potentially attractive/unattractive strains, we first gave a choice between wild-type (HNI) and *b*^*g8 *^females to males of various strains (Figure 2a). The body color of HNI is brown due to black melanophores and orange xanthophores in the skin. The color of *b*^*g8 *^is orange due to colorless melanophores and pigmented xanthophores [18]. Although medaka with eight *opsin *genes [19] and humans with three *opsin *genes may sense colors differently, we assume that medaka can at least differentiate these and other body colors (Figure 1). Some, but not all, of the males reproducibly preferred *b*^*g8 *^females, which made the overall male approaches weakly biased from 50:50. However, we are hesitant to conclude from this result that the color of *b*^*g8 *^is more attractive than that of HNI (see also Figure 3a). This is because we observed that the *b*^*g8 *^females swam about in the tank more actively than the inbred HNI females, which may have attracted more interest from the males (similarly, the HNI males were less vigorous (25 ± 3.7 approaches/hour) in comparison with males of other non-inbred strains; see above).

The females spawned 110 times in total, most of which (78% = 86/110) took place within five male approaches (Figure 2b). It was surprising to us that the females mated with the grossly unnatural red/green (OlMA1-DsRed2/GFP) transgenic males without any hindrance even on the first day of the experiments. Nevertheless, a potential sign of sexual discrimination could be detected from the *b*^*g8 *^females mating with *ci *males that had gray skin due to decreased xanthophores and increased white leucophores [20]; that is, the *b*^*g8 *^females spawned within 3.7 ± 0.68 approaches with other males, but claimed 18 ± 5.1 approaches from the *ci *males (one-way ANOVA, *F*_4,45 _= 7.059, *P *< 0.001). On the contrary, the HNI females did not show such discrimination against the identical *ci *males (spawned within 1.9 ± 0.3 and 3.3 ± 0.6 approaches with *ci *and other males, respectively; one-way ANOVA, *F*_5,55 _= 2.030, *P *= 0.089). This demonstrates that the delays in mating between the *b*^*g8 *^females and the *ci *males are due to *b*^*g8 *^females' choice rather than *ci *males' inability or lack of interest in mating (note that the *b*^*g8 *^females were even more frequently approached by the *ci *males than the HNI females; Figure 2a). The difference in choosiness between the *b*^*g8 *^and HNI females is interesting, but the cause remains unknown (possibly, less choosy HNI females might be selected during establishment of this inbred strain, because of the sexually unenthusiastic males; see above).

**Figure 4:**
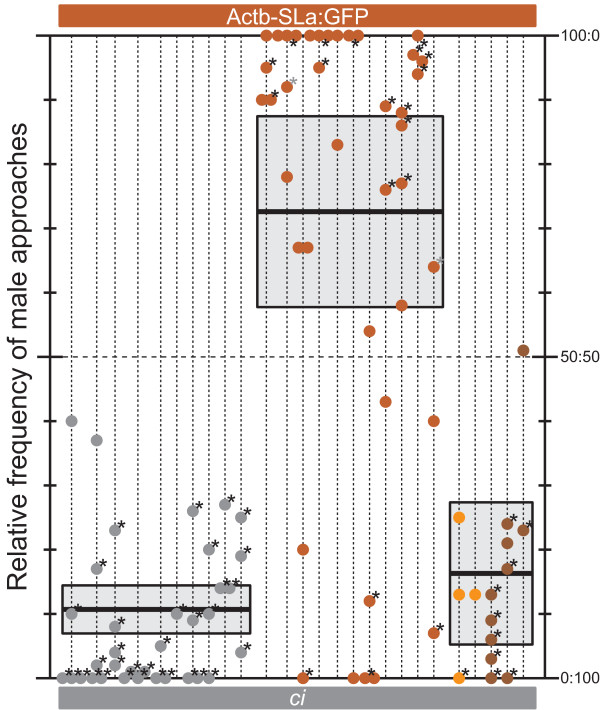
**Harmonious effects of *SLa *expression on secondary sexual characters and mating preferences**. Male mating preferences (*n *= 20) between *ci *and Actb-SLa:GFP females (*n *= 8; 4 for each strain). Note that *ci *males' maximally biased preference towards *ci *females is reversed in Actb-SLa:GFP males. Actb-SLa:GFP is only a single-gene switch from *ci *[23].

We then asked whether or not *ci *females are similarly unattractive to reproductive partners (Figure 2c). We offered each male a choice between all six combinations of *b*^*g8*^, *ci*, OlMA1-DsRed2, and OlMA1-GFP females, and detected non-random male approaches only when one of the choice females was *ci*; that is, the males preferred the other choice regardless of her color. Interestingly, *ci *males occasionally, and with the exception of others, seemed to prefer *ci *females (see also Figure 4), whereas males of other strains seemed not to distinguish females of the same and different strains. Indeed, medaka males cannot even distinguish con-specific (*O. latipes*) and hetero-specific (*Oryzias curvinotus*, *Oryzias luzonensis*, or *Oryzias celebensis*) females in spite of severe hybrid incompatibilities (embryonic lethality or adult sterility (see [21]); our unpublished observation). Therefore, the sexual discrimination against *ci *(Figures 2b and 2c) is a unique phenomenon. Something indispensable for mate attraction, which can be regarded as secondary sexual characters in this species, must be lost from *ci*.

### Sexual discrimination of males against *ci *females under various conditions

Focusing on the *ci *female's unattractiveness (Figure 2c), we designed two sets of experiments to further dissect this phenomenon. As already described, the body-color defect of *ci *consists of two major components: decreased xanthophores and increased leucophores. Taking advantage of a *ci*-*leucophore free *(*lf*) double-mutant strain that has decreased xanthophores but no leucophores [22], we revealed that the decreased xanthophores are sufficient to cause the unattractiveness; that is, *ci*-*lf *females were similarly unattractive to males as *ci *females (Figure 3a).

Next, we offered males a choice between *b*^*g8 *^and *ci *females either under the free-swimming condition, in flasks, or in holed flasks. When the choice females were confined to the flasks, the number of male approaches (more precisely, the number of male behaviors we scored as an approach, which (particularly the positioning under a female) was actually interrupted by the wall of the flasks; 23 ± 2.1 approaches/hour) was strikingly reduced in comparison with that under the free-swimming condition (64 ± 4.5 approaches/hour). The number further decreased when females were in the holed flasks (10 ± 1.4 approaches/hour), presumably because the holed walls obscured the image of females. Nevertheless, the males preferred *b*^*g8 *^to *ci *under all three conditions (Figure 3b).

Thus, the sensory cue causing the male's sexual discrimination against *ci *is transmittable through the non-holed transparent plastic wall (that is, olfactory/electric cues excluded). Although auditory/behavioral/magnetic cues may also participate, it is most likely that visual cues from xanthophores play a crucial role as secondary sexual characters in medaka.

### Effects of *somatolactin alpha *on the secondary sexual characters and mating preferences

Whatever the actual sensory cues are, the sexual unattractiveness of *ci *must have its roots in a mutation in the genome. And here, the medaka system can best show its excellence. The *ci *mutation has been identified on a gene encoding somatolactin alpha (SLa), the closest relative of growth hormone in fish [20]. Therefore, we predicted that transgenic *SLa *expression would rescue the unattractiveness of *ci*, and that stronger expression would make the fish more attractive. Based on these assumptions, we established a transgenic *ci *strain that ectopically over-expresses *SLa *under the control of the *β-actin *(*Actb*) promoter, the Actb-SLa:GFP strain [23].

As expected, the secondary sexual characters (xanthophores) were dramatically enhanced in Actb-SLa:GFP [23]. We offered males a choice between the unattractive *ci *and the presumably hyper-attractive Actb-SLa:GFP females. Age, genomic background, environmental conditions, and phenotypes other than the body color of the choice females were strictly standardized prior to the experiments; that is, we used siblings between *ci *and hemizygous Actb-SLa:GFP which were born in the same week, bred in the same tank, and size-matched as best as we could in both length (from the snout to a distal edge of the caudal fin) and weight of the body (*ci*, 32.8 ± 0.25 mm and 356 ± 16 mg; Actb-SLa:GFP, 34.0 ± 0.91 mm and 400 mg ± 19 mg; *P *= 0.235 and 0.124, respectively).

The result was partly unexpected but exciting (Figure 4). First, male mating preferences were biased to the utmost; as if there was only one female in the tank, the males often single-mindedly approached one of the two choices (max. 86 approaches/hour) while the other female was completely ignored. Second, the extremely strong preferences were biased in the opposite direction between the mutant and transgenic males; that is, while the Actb-SLa:GFP males preferred to mate with the Actb-SLa:GFP females, the identical Actb-SLa:GFP females were never preferred by the *ci *males. Thus, the *SLa *expression not only increased xanthophores but also made the fish prefer their mates to have increased xanthophores (that is, the harmonious changes of secondary sexual characters and mating preferences; see *Background*). Third, males of other strains (that are wild-type for the *ci *locus) unexpectedly preferred *ci *to Actb-SLa:GFP; that is, the over-expression of *SLa *made *ci *even less attractive for the wild-type males.

Although interpretation of the third result is difficult (discussed below), all the present results consistently support the conclusion that expression of *SLa*, but not other genes tested in this study (that is, *DsRed*, *GFP*, *b*^*g8 *^[*Slc45a2*], *i*^3 ^[*Pink-eyed dilution*], and *lf*), crucially affects sexual attractiveness and biases mate-choice behaviors.

## Discussion

### The medaka model for mate-choice studies

One important finding in this study is the fact that this model organism for functional genetics apparently chooses reproductive partners (Figures 3 and 4). This medaka system should therefore provide precious opportunities for studying mate-choice behaviors at both the organismal and molecular levels, as partly demonstrated in this study.

The mate-choice experiments we conducted are different from the classical three-compartment method where one test female is placed between two choice males in neighboring tanks. Assessing female association preferences under this restricted physical contact is logical, because females are generally choosier than males (that is, significant preferences can be more easily detected) and choice males often fight for a test female (that is, her final mating decision under free-swimming conditions may only reflect dominance hierarchy of the males). Medaka has also been studied by this method, and Howard *et al*. [24] concluded that association preferences of a female could be reflected in her mating decision only when competition between males is weak. Therefore, the free-swimming method using one test male and two (or more) choice females [14] would provide a simpler and more sensitive (see Figure 3b; [25]) but still biologically significant platform (that is, if female preferences are not reflected in mating, assessing female preferences would have little biological meaning) for studying mate choice in this species.

### Rapid evolution of pre-mating sexual isolation by a single mutation

Another important finding in this study is the gene that distinctly affects the mate-choice behaviors, *SLa*. Male mating preferences were maximally biased in the choice between *ci *and Actb-SLa:GFP (Figure 4), most likely via visual cues from xanthophores (Figure 3). Furthermore, the maximally biased mating preferences were opposite in direction between the *ci *and Actb-SLa:GFP males (Figure 4). These results are the first demonstration of a single-gene expression harmoniously changing both secondary sexual characters and mating preferences (see *Background*).

This finding may fulfill a very important prediction made by Fisher [26]. He proposed that secondary sexual characters and mating preferences come to be genetically correlated as a consequence of choice itself. This 'runaway' mechanism of sexual selection has already been supported by many theoretical and limited empirical studies proposing the joint evolution of two or more genes that control either secondary sexual characters or mating preferences [10-12,27,28]. Our present results (Figure 4), however, may provide the best and simplest example of Fisherian evolution. The genetic correlation between secondary sexual characters and mating preferences (that is, a positive feedback of intra-variant mating) can be established at the highest speed (within one generation) by switching on (Actb-SLa:GFP) or off (*ci*) a single gene. By contrast, other theories of 'adaptive' sexual selection (for example, the good genes, reinforcement, and so on (see [28])) do not explain the present results, because the genomes of *ci *and Actb-SLa:GFP differ solely by the *SLa *transgene and the strains are, of course, post-zygotically fully compatible.

We speculate that SLa and its up/downstream cascades could be interesting targets for uncovering the molecular basis of visual (body color)-based mate choice in wild populations. This is because some fish species up-regulate SLa during reproductive seasons [29,30], and xanthophores (carotenoids) are generally sensed as preferable secondary sexual characters [5]. Furthermore, assortative mating sometimes involves carotenoid-based (yellow-red) and structural-based (blue-gray) colorations [31,32], which are rather similar at a cellular level to the xanthophore-dominant Actb-SLa:GFP and irridophore-dominant *ci *medaka [23]. In contrast, visual-based mate choices in higher vertebrates must utilize different genetic mechanisms, because *SLa *has been lost from tetrapods during evolution [33].

### Mechanisms through which *SLa *shapes mating preferences

Another important direction for future studies is to investigate how *SLa *functions as a gene for mating preferences (see *Background*). The mating preferences symmetrically biased between *ci *and Actb-SLa:GFP (Figure 4) should provide an ideal opportunity for this purpose. Three working hypotheses are conceivable at the moment. First, SLa directly affects neural circuits/activities in the brain or sensory organs (eyes), which can be supported by the broad expression of *SLa receptor *(*SLR*) [34]. Second, medaka shapes mating preferences based on its own body color. This indirect action of SLa seems to more plausibly explain the harmonious change of secondary sexual characters and mating preferences (this self-referent behavior, however, may not explain the runaway evolution of sex-specific ornaments which the model was originally aimed to explain; see above). Third, medaka shapes mating preferences based on the color of tank mates which they grew up with (see *Methods*). Considering that this potential familiarization/learning is apparent only in the *ci *and Actb-SLa:GFP males (Figures 2c and 4), this scenario still supports the distinctive role of SLa (but not the red/green fluorescent proteins) in shaping mating preferences. All these hypotheses could be addressed in the medaka system; for example, experiments using various tank mates, the *eyeless *or xanthophore mutants other than *ci *[35,36], diet (carotenoid) restriction, or *SLR *knockout.

It is also important to investigate why the wild-type males preferred *ci *to Actb-SLa:GFP (Figure 4). We speculate that neither too high nor too low but only optimal expression of SLa (that is, optimal level of xanthophore distribution in the skin) can make medaka most attractive for the wild-type males (see [37,38]). Alternatively, Actb-SLa:GFP females may have a deleterious side effect due to the ectopic over-expression of *SLa*, which we have not yet identified. If the latter were the case, however, mating preferences could not have been symmetrically biased between the *ci *and Actb-SLa:GFP males; that is, Actb-SLa:GFP females should have become unattractive even for Actb-SLa:GFP males. Additional *SLa*-transgenic *ci *strains with a series of promoters weaker than *Actb *would help to address this question.

Thus, the present finding of the *SLa*-dependent mate choice enables many ingenious experiments to be designed in this and other fish species. The medaka system, with excellent tools for genomic experiments [39], should further facilitate molecular dissection/manipulation of visual-based mate choice. Comparison of genetic mechanisms for the visual/olfactory/auditory-based mate choices (see *Background*) will steadily uncover how the divergent tactics in sexual selection have evolved.

## Conclusion

Taken together, we have: (1) established systems to evaluate mating preferences in medaka, (2) screened the distinctively unattractive *ci *mutant, (3) identified the secondary sexual characters as xanthophores in the skin, (4) verified by genetic engineering that the gene mutated in *ci *(*SLa*) controls sexual attractiveness, and (5) found that mating preferences are also affected (directly or indirectly) by *SLa *expression. The dual control of secondary sexual characters and mating preferences by *SLa *should argue that one mutation on a single gene could be sufficient to facilitate runaway evolution of assortative mating that would promote sympatric speciation.

## Methods

### Fish and breeding conditions

All the fish were hatched and bred in the laboratory *en masse *(each strain separately, except that the *ci *and Actb-SLa:GFP sibling fish were kept together until their phenotypes became obvious (~1 month after hatch)). When fish had reached sexual maturity, one male and two females were placed into a tank (20 × 13 cm^2 ^with a water level of ~5 cm), which was also used for mate-choice experiments, and reared until they spawned eggs daily. The water temperature was ~28°C and light was provided solely by ordinary fluorescent lamps for 14 hours per day (8:30 to 22:30).

### Mate-choice experiments

We set up four tanks of one male and two females, which were kept separated by translucent plastic dividers with several slits. The next morning, we removed the dividers and video-recorded their behavior from above for one hour between 9:00 and 11:00. Between the neighboring tanks, we placed brown cardboard to avoid visual contact. After the experiments we separated the male and females again by the dividers and rotationally moved males to other tanks for experiments the next morning. We continued these experiments using the same fish (four males and eight females) in different combinations for four (or more) consecutive days. When finished, we replaced all the fish and conducted another set of consecutive experiments. When daily spawning females of similar sizes were not available, we used the same females but in different combinations.

For the non-free-swimming experiments (Figure 3b), we used cell culture flasks (CELLSTAR; Greiner bio-one) for partitioning the tank. Eighteen holes of 4 mm in diameter were bored into the flasks, when necessary. We manually analyzed the recorded behaviors using iMovie software (Apple).

### Statistics

We independently calculated 95% confidence limits of relative frequencies of male approaches for each female combination to find out whether the limits include 50:50 (that is, no choice). For this purpose, we first summed the number of approaches for each male (that is, a group of four or more mate-choice results shown on each vertical dotted line in Figures 2, 3, and 4) and calculated the relative frequencies for each male. We also independently calculated *P *values of each mate-choice result by the two-tailed binomial test to find how significantly the male approaches are biased from 50:50.

To the data in Figure 3a we also applied the one-way repeated measures ANOVA to compare means of the relative frequencies under the three different conditions (that is, female combinations of *b*^*g8*^-HNI, *b*^*g8*^-*ci*, and *b*^*g8*^-*ci*-*lf*). For the data in Figure 2c, we also applied the one-way repeated measures ANOVA. For this purpose, we summed the relative frequencies of male approaches toward each of the four female strains and used the values (that is, min. 0% and max. 300%, because each female strain was used in three combinations) as variables showing females' attractiveness.

## Authors' contributions

SF conceived/conducted the mate-choice experiments, analyzed the video-recorded behaviors, performed the statistical analyses, and wrote the manuscript. MK established the Actb-SLa:GFP transgenic medaka. KA and SO participated in the behavioral analyses and coordination/management of the experiments, respectively. AM and HM provided all the spaces and running costs for the experiments in Germany and Japan. All authors read and approved the final manuscript.

## Supplementary Material

Additional file 1 **Medaka mate-choice behaviors**. A *b*^*g8 *^male is given a choice between females of OlMA1-DsRed2 and OlMA1-GFP. Click here for file

Additional file 2 **Medaka spawning**. An OlMA1-GFP male is given a choice between females of OlMA1-DsRed2 and OlMA1-GFP. Click here for file
